# Supplementary material for: Content-rich biological network constructed by mining PubMed abstracts
Source: BMC Bioinformatics. 2004 Oct 8;5:147. doi: 10.1186/1471-2105-5-147 (PMC528731; doi:10.1186/1471-2105-5-147)
Supplement: Additional File 2 — The original results of the above study (non-essential files are deleted to keep the file size under the limit set by BMC bioinformatics). [file 1471-2105-5-147-S2.bz2 › chilibotAdditionalFile2/dip20/575ID9204896E10943/html/left.html]

 


### Chilibot Session: 575ID9204896E10943

|  |  |  |  |
| --- | --- | --- | --- |
| Home | New Session | Folders | Log Out |

View legend |
View input file |
Query history |
Image source file

that are above sentence level
that are interactive
with weight > 1
with weight > 2
with weight > 3
with weight > 4
with weight > 5


---

**Statistics:**  

```
Searches performed: 1  
Relevant PubMed records: 178  
PubMed records processed: 20 (11.23%)  
Number of links found: 1   

Start  time: Sun Aug  3 19:49:36 2003   
Finish time: Sun Aug  3 19:49:42 2003
```

---

**Solitary terms:** None
